# Optical coherence tomography attenuation imaging for lipid core detection: an ex-vivo validation study

**DOI:** 10.1007/s10554-016-0968-z

**Published:** 2016-09-12

**Authors:** Muthukaruppan Gnanadesigan, Ali S. Hussain, Stephen White, Simon Scoltock, Andreas Baumbach, Antonius F. W. van der Steen, Evelyn Regar, Thomas W. Johnson, Gijs van Soest

**Affiliations:** 1 Department of Biomedical Engineering, Erasmus Medical Center, PO Box 2040, 3000 Rotterdam, CA The Netherlands; 2 Bristol Heart Institute, Bristol, UK; 3 School of Clinical Sciences, University of Bristol, Bristol, UK; 4 Department of Imaging Science and Technology, Delft University of Technology, Lorentzweg 1, 2628 Delft, CJ The Netherlands; 5 Thorax Center, Erasmus Medical Center, PO Box 2040, 3000 Rotterdam, CA The Netherlands

**Keywords:** Optical coherence tomography, Attenuation, Lipid core plaque

## Abstract

Lipid-core atherosclerotic plaques are associated with disease progression, procedural complications, and cardiac events. Coronary plaque lipid can be quantified in optical coherence tomography (OCT) pullbacks by measurement of lipid arcs and lipid lengths; parameters frequently used in clinical research, but labor intensive and subjective to analyse. In this study, we investigated the ability of quantitative attenuation, derived from intravascular OCT, to detect plaque lipid. Lipid cores are associated with a high attenuation coefficient. We compared the index of plaque attenuation (IPA), a local quantitative measure of attenuation, to the manually measured lipid score (arc and length) on OCT images, and to the plaque characterization ex-vivo. We confirmed a correlation between the IPA and lipid scores (r^2^ > 0.7). Comparison to histology shows that high attenuation is associated with fibroatheroma, but also with macrophage presence. IPA is a robust, reproducible, and user-independent measure that facilitates quantification of coronary lipid, a potential tool in clinical research and in guiding percutaneous coronary intervention.

## Introduction

Sudden rupture of a lipid-rich atherosclerotic plaque, causing acute myocardial infarction is one of the major causes of death worldwide. Early detection of such coronary plaques may influence treatment strategies and facilitate a reduction in clinical events secondary to ischemic heart disease [1]. The lipid core plaque (LCP) or fibroatheroma is a type of atherosclerotic lesion prone to develop unstable features under the influence of inflammatory processes and mechanical forces. Hence, imaging of tissue composition, especially in LCP, plays an important role in recognizing plaque instability [2]. LCP detection also has implications in the guidance of coronary interventions, as these plaques have been implicated in peri-procedural and follow-up events [3–8]. Intravascular optical coherence tomography (OCT) is now widely used as a clinical tool, imaging vessel lumen and wall morphology for guidance of stent placement and optimization [9–12]. OCT is a catheter-based imaging technique that provides high-resolution images of the arterial wall. Interpretation of the images allows for a qualitative assessment of the tissue composition [7, 11, 12].

Measurement of lipid length and lipid arc in OCT for calculation of a lipid score is a common method to quantify lipid in cardiovascular research [13, 14]. This score is an indicator of the extent and severity of atherosclerotic disease in the coronary arteries. It provides patient and lesion-specific diagnostic information, and may serve as a metric of plaque progression in temporal studies. Currently a skilled OCT reader is required to score lipid accumulations, and it is a time-consuming manual procedure with considerable inter-observer variability [13]. An automated tissue score that can provide the same information can be a very useful tool in research with potential for clinical utility.

Various tissue components have different optical parameters and this contrast can be exploited to devise a tissue characterization method based on IV-OCT data [15]. Quantification of tissue optical parameters may assist image interpretation by OCT users. The attenuation coefficient is a robust tissue optical parameter [16] that has been proposed for tissue characterization [17–19]. Lipid-rich necrotic core and macrophage infiltration have high attenuation compared to fibrous tissue and other plaque components. Such methods have recently been augmented with statistical image metrics to achieve tissue detection [20].

In this study, we validate optical attenuation imaging of coronary plaque tissue type in an ex-vivo setting, using whole heart specimens harvested from cadavers and imaged in a purpose-built setup. We focus on the ability of attenuation imaging to identify coronary plaque lipid. The objective of the study was to compare the quantitative attenuation scores with the manually measured lipid scores of plaque segments, which were characterized based on the histology.

## Materials and methods

### Specimens

Whole cadaveric hearts used were obtained from the Bristol Heart Valve Bank and excised within 48 h post-mortem and stored at 4 °C [21]. Hearts were randomly selected from suitable specimens; meaning the major conduit coronary arteries needed to be intact. A short guide catheter was introduced into the right coronary artery to facilitate intravascular imaging and fixed in position with sutures. All tissues were handled in accordance with the local ethics regulations. The heart specimen was held within a custom-built Perspex container, with adapters on both sides of the lid allowing connection of the guide internally, and a Y-connector and pressure/injector manifold externally [21]. For the study a total of six heart specimens were imaged.

### IV-OCT imaging

For intravascular OCT imaging, the imaged artery was perfused with phosphate buffered saline (PBS) and the intracoronary pressure was maintained at 100 mmHg. The OCT image pullbacks of the coronary arteries were performed at 20 mm/s. The OCT system used for imaging was a C7-XR with Dragonfly catheter (St. Jude Medical Inc. St. Paul, MN, USA).The end of the guide catheter served as a reference point.

### Histology and plaque characterization

The imaged vessels were pressure-fixed while still on the heart with buffered formalin at 100 mmHg for 15 min, excised from the heart, and fixed with buffered formalin for 24 h. Then the vessels were embedded in paraffin and cut into 4-mm blocks with the end of the guide catheter serving as a reference. The proximal face of each block was cut at 3 µm thickness for histological analysis and serial sections stained with Haematoxylin and Eosin (H&E), Elastic Van Gieson (EVG), Cluster of Differentiation 68 (CD68) and smooth muscle cell α-actin stains. CD68 and α-actin were visualized using mouse anti-CD68 (DAKO M0814 and M0876 mixed 1/200 of each), and mouse anti-α actin (DAKO, M0851 1/200), or matched mouse IgG control, followed by biotinylated goat anti-mouse and extravidin-HRP conjugate with DAB staining.

Twenty-three atherosclerotic plaques were identified from the six cadaveric heart specimens. A skilled pathologist analysed the plaque characteristics by the histological staining and classified the plaque type. The lesions were classified into pathological intimal thickening, pathological intimal thickening with macrophage infiltration and fibroatheroma using standard criteria [2, 22]. Fibroatheroma with less than 65 μm cap thickness was classified as thin-cap fibroatheroma.

### Parametric imaging

**Fig. 1:**
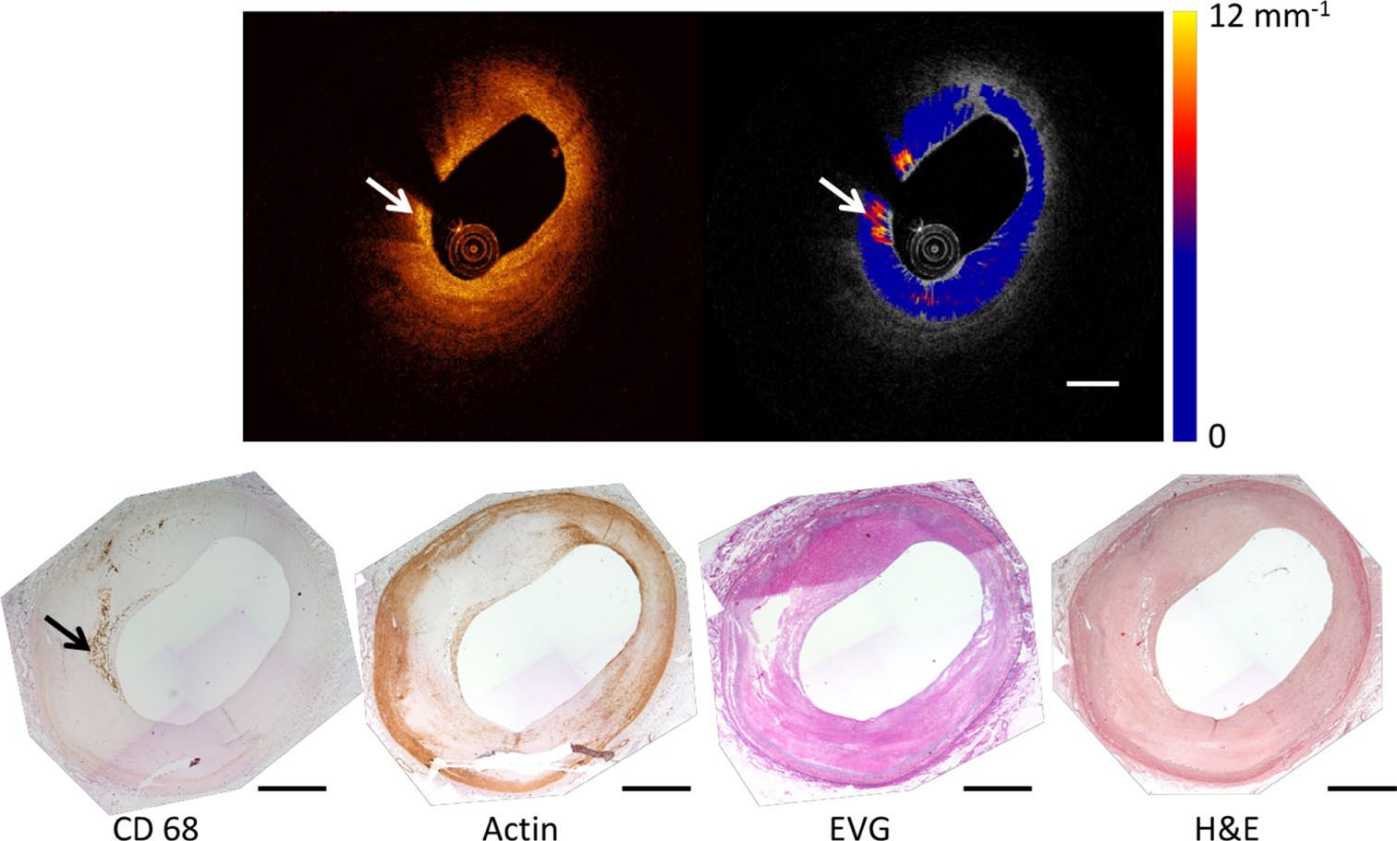
A representative OCT frame and the corresponding attenuation image depicting high attenuation features and the corresponding histology stains. The *arrow heads* point to a streak of macrophages that shows high attenuation and are clearly stained in CD68. The *dashed scale bar* equals 1 mm

The acquired OCT images were analysed to quantify the attenuation coefficient of the tissues by fitting the OCT signal to a single scattering model [18, 19].

1 $$\left\langle {{\text{I}}_{{\text{d}}} } \right\rangle = {\text{T}}\left( {\text{r}} \right){\text{S}}\left( {\text{r}} \right){\text{I}}_{{\text{0}}} {\text{e}}^{{ - \mu_{{\text{t}}} {\text{r}}}}$$

where S(r) is the OCT signal roll off and T(r) is the point spread function (PSF) of the catheter [23]. The attenuation coefficient *µ*_t_ is the parameter of interest. The attenuation calculation and the model implementation were done in Matlab release 2012b (The Mathworks, Inc., Natick, MA, USA). The model is fitted in the polar image, in every A-line starting from the edge of the lumen [18, 20], in small windows of varying length [24] to extract the attenuation coefficients. The accuracy of the extracted attenuation coefficient is approximately 1 mm^−1^ [18]. The data analysis results in an attenuation image per frame of the pullback. Figure 1 depicts a frame of OCT, its corresponding attenuation image and histology.

**Fig. 2:**
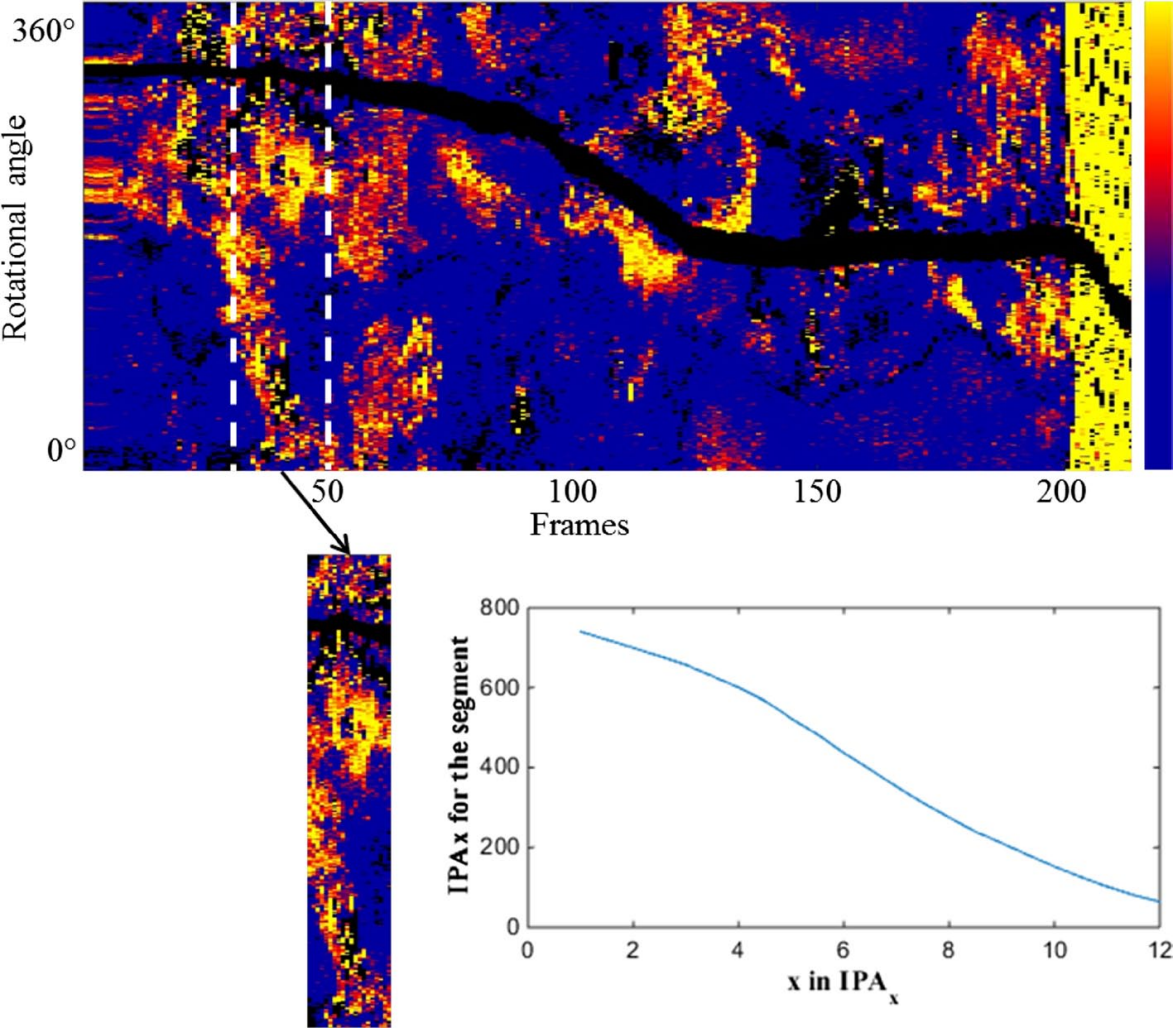
A representative image of the en-face map depicting the attenuation features across the vessel and the 4 mm segment of a plaque that would be used to calculate the IPA. The *inset plot* shows the IPA for the segment for different thresholds ‘*x*’ in IPA_*x*_. *Color scale* runs from 0 to 12 mm^−1^

We made longitudinal attenuation maps of the vessel depicting the tissue properties of the intima along the entire pullback. The map was constructed by sampling the maximum value of attenuation between the lumen border and the internal elastic lamina (IEL) as shown in Fig. 2. The en-face map display has dimensions of frame number (horizontal-axis), rotational angle (vertical-axis) and color-codes the maximum attenuation coefficient per A-line in the range 0–12 mm^−1^. Such a map highlights strongly attenuating features like the lipid plaques in the entire pullback [16] and corresponds well with a visual assessment of LCP in the OCT data by an expert reader [25].

We also defined an Index of Plaque Attenuation (IPA) to quantify the parametric image. IPA is the ratio of the number of pixels in the attenuation map or a segment of it with an attenuation coefficient greater than a certain threshold *x* to the total number of pixels, multiplied by a factor of 1000. Mathematically it is represented as

2 $$IPA_{x} = \frac{{N(\mu _{t}> x)}}{{N_{{total}} }} \times 1000$$

where *N* is the number of pixels. *x* is the threshold in attenuation coefficient, with a maximum value of 12 mm^−1^, that enables IPA to represent a particular tissue type.

The IPA was calculated in 4 mm windows, covering a plaque segment. For the frame pitch of 200 µm that we used in our study, a window for IPA computation comprises 20 OCT frames. The OCT data were matched to the histology cross-sections based on longitudinal position and general anatomy (side branches and presence of plaque).

### Lipid measurements

Lipid measurements were made on the OCT frames, using the Ilumien offline OCT review workstation by St. Jude Medical (St. Paul, MN, USA). The lipid length was measured by detection of sequential OCT frames within a plaque segment containing lipid, defined as a diffusely bordered signal-poor region with high attenuation by the signal rich region covering them [7, 26]. The pitch of the OCT enabled calculation of the lipid length in mm. The lipid arc measurements were made by defining the circumferential extent of the lipid core from the vessel centre [14] and a mean for the plaque segment was calculated. Combining both measurements a lipid score was calculated, which is the product of the average lipid arc of the segment and the lipid length of the segment. To establish the relation between OCT lipid score and optical attenuation coefficient, we computed the correlation (Pearson’s *r*) between lipid score and IPA_*x*_ for different values of *x* (1, 1.5, …, 12) in all segments.

### Statistical analysis

Continuous variables are expressed as mean (standard deviation), or median (interquartile range; IQR) and categorical variables are expressed as percentages. The regression analysis was performed on the lipid measurements and IPA, with linear least squares fit and the coefficient of determination *r* ^2^ (square of the correlation coefficient *r*) was used.

## Results

According to the analysis of the histological sections, there were 16 fibroatheromas, 3 fibrous plaques, 2 pathological intimal thickenings and 2 pathological intimal thickenings with macrophage infiltration. One fibroatheroma with <65 microns cap thickness was classified as thin cap fibroatheroma.

We measured the lipid scores in the OCT recordings of the segments of interest. The median of the mean lipid arc of the plaques was 81.98 ± 45 degrees and the median of the lipid length was 3.9 ± 2.1 mm. The scores ranged from 0 to 638.4 mm °. There were eight plaques that did not have the characteristics of LCP according to the consensus OCT criteria [7].

**Fig. 3:**
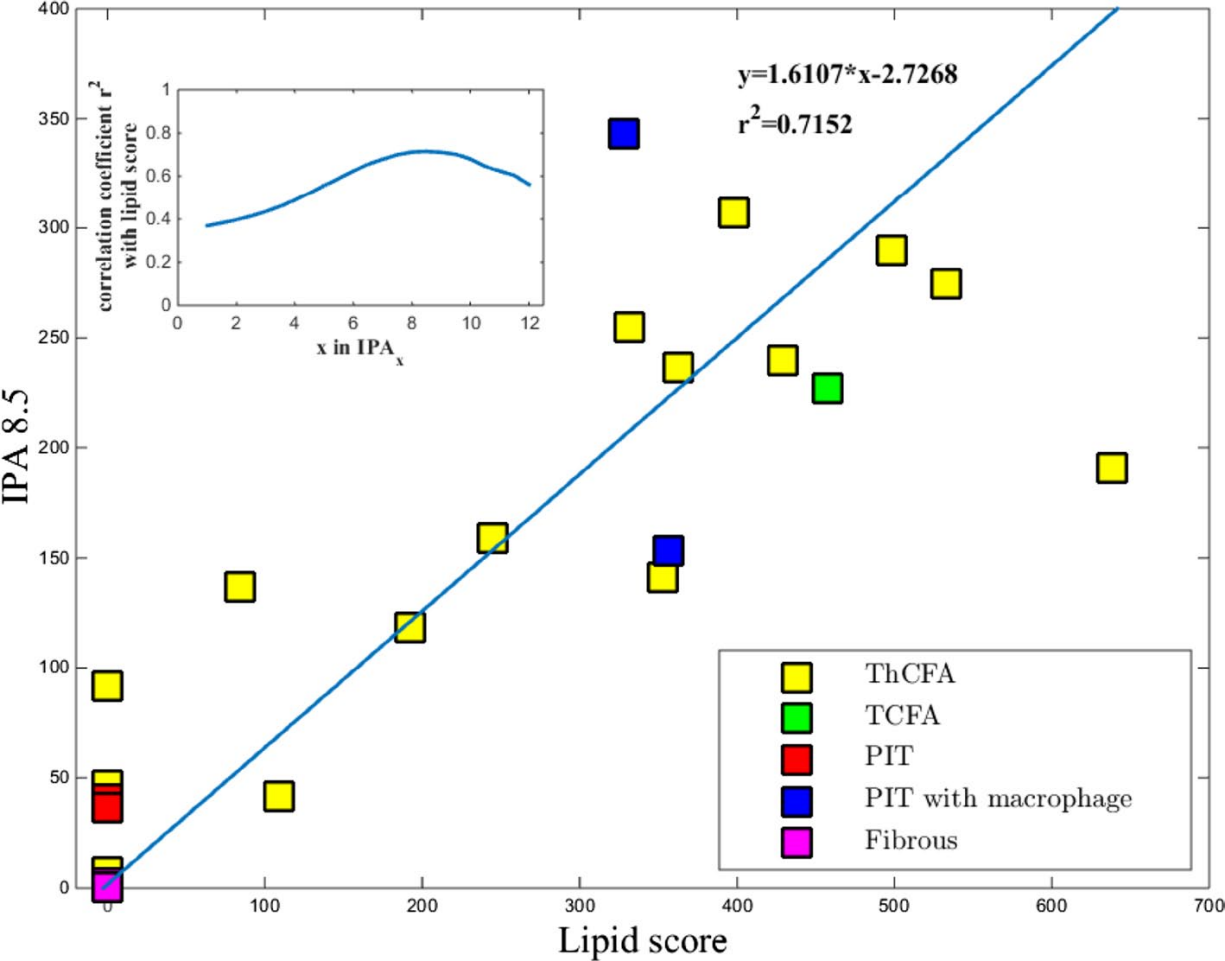
Correlation between IPA8.5 and lipid score by OCT (the product of mean lipid arc and lipid length). The *inset plot* shows the correlation coefficient with the lipid score for different thresholds ‘*x*’ in IPA_*x*_. The *legend* shows the colour code for plaque types

The maximum correlation coefficient between the lipid score and IPA_x_ was found for a threshold of *x* = 8.5 mm^−1^ (IPA_8.5_), and equalled *r* = 0.85 (p < 0.0001). The relationship between IPA and the lipid scores for all the 23 plaques are given in Fig. 3. The data points in the graph are color-coded based on the plaque type classification as shown in the legend.

## Discussion

**Fig. 4:**
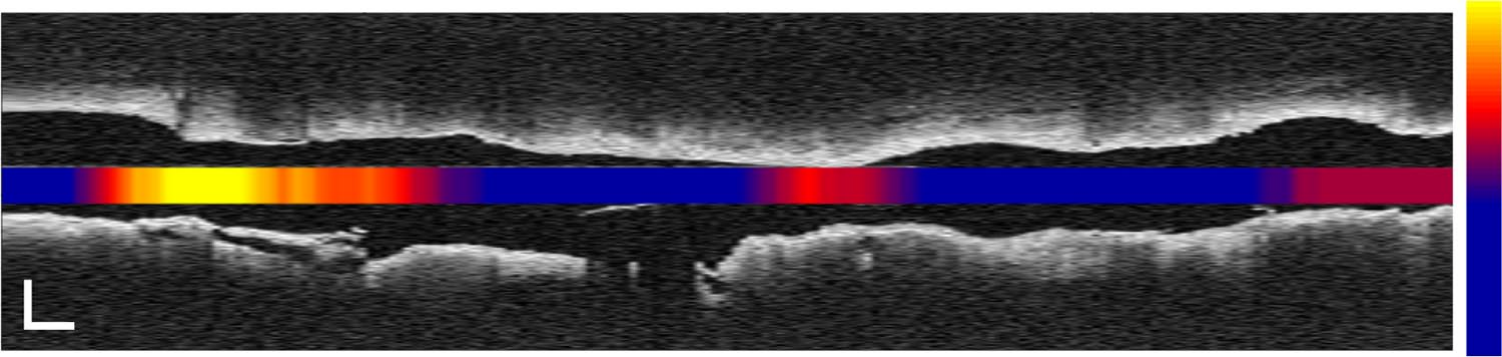
Colour coded IPA_8.5_ embedded in an OCT L-mode image of an artery, showing high IPA at a site of plaque rupture. *Colour scale* runs from 0 to 300; *scale bars* represent 1 mm

This study investigated the ability of the index of plaque attenuation (IPA), a bias free and reproducible summary measurement of attenuating tissue types, to detect lipids. The study aimed to validate OCT attenuation coefficient as a lipid-core detection tool. The main finding of the study was that the OCT-derived index, the IPA_8.5_, have a significant correlation (*r* ^2^ > 0.7) with the manual lipid score in OCT images, which enables automation of the coronary plaque lipid assessment by OCT. Lipid scoring is a time consuming process, where frame-by-frame analysis and measurement is required. Previous studies which compared the lipid score by OCT to automated indices like the near-infrared spectroscopy (NIRS)-based Lipid Core Burden Index (LCBI) found a lesser correlation (*r* ^2^ = 0.436) [27]. This weaker association between OCT measurements and LCBI may be explained by registration errors in measurements made by different catheters, but also by the physically different detection mechanisms (scattering-dominated attenuation for OCT, vs. optical absorption in NIRS). The IPA, which is the result of automatic computation of plaque attenuation, has the potential as a clinical tool as it can indicate the presence and location of lipid rich plaques in an entire pullback, and provides an alternative to manual scoring in cardiovascular research. IPA could be displayed as a color-coded block along the vessel indicating probable lipid core plaques as shown in Fig. 4. IPA, being an index calculated from a physical parameter, is robust and reproducible.

Figure 3 demonstrated that thick cap fibroatheroma exhibited a range of low to high IPA values, showing that lipid content is just one tissue component contributing to optical attenuation. We only found one thin cap fibroatheroma in our data, which had large OCT-lipid burden as expected [14]. It had a high IPA value, illustrating the relation between manual OCT measurements and automatic IPA analysis. Its typical dense macrophage infiltration by histology contributed to the observed attenuation. Among the four plaques classified as pathological intimal thickening (PIT), two had macrophage infiltration. The two PITs with macrophage exhibited high IPA values even though pathological evidence of lipid was not available. The gap between the PITs with and without macrophages was very significant, as expected. The three fibrotic plaques had near zero IPA values, as expected. The three fibrous plaques and the four PITs were among the eight plaques scored as having no lipid on OCT, along with one fibroatheroma with deep extra-cellular cholesterol.

### Limitations

Our data set is targeted towards validation of lipid measurements, and so does not reflect the heterogeneity of atherosclerosis in a clinical setting, with few plaques other than FA, and only one TCFA. The two included macrophage-infiltrated PIT plaques exhibit high attenuation even in the absence of histological evidence for lipids, a known mechanism resulting in false positives for lipid detection by OCT [7, 28, 29]. It was not possible to compare IPA with the lipid scores from histology as it is not practical to measure lipid length and the mean lipid arc in the whole segment. The IPA is effectively an area measure, which can be affected by the position of the catheter relative to the centre of the lumen, as is the LCBI based on NIRS. For small to moderately sized vessels, the difference is smaller than the scatter in the data we observe in Fig. 3. We did not apply a correction in this analysis, although it would be possible in principle by remapping the attenuation analysis to coordinates relative to the lumen center. The attenuation calculation itself is automated and fast but in calculation of IPA, manual media segmentation is currently necessary to avoid artefacts due to the intima-media border. To be able to automate the whole process and for application to large datasets, automated media segmentation is required. This software is currently under development in our institute [30].

## Conclusion

We validated the optical attenuation coefficient, as measured by OCT, as a tissue classification tool in atherosclerotic plaques. We investigated a quantitative measure of the attenuating tissue types, the index of plaque attenuation (IPA). Our results show that the OCT-derived IPA_8.5_, quantifying the fraction of vessel wall with a maximum *µ*_t_ > 8.5 mm^−1^, has a correlation (*r* ^2^ > 0.7) with the conventional lipid score on OCT images. The results highlight the potential of a robust and fast identification tool of lipids in OCT pullbacks.
